# Lenalidomide and Dexamethasone for a Patient of POEMS Syndrome Presenting with Massive Ascites

**DOI:** 10.1155/2014/818946

**Published:** 2014-02-19

**Authors:** Shuji Ueda, Sayoko Yonemoto, Kazumasa Oka, Naohiko Fujii, Keiichi Nakata, Hitomi Matsunaga, Seiko Kataoka, Yuki Iwama, Hiroyuki Narahara, Yuichi Yasunaga, Yoshiaki Inui, Sumio Kawata

**Affiliations:** ^1^Department of Internal Medicine, Hyogo Prefectural Nishinomiya Hospital, 13-9 Rokutanji-cho, Nishinomiya, Hyogo 662-0918, Japan; ^2^Department of Pathology, Hyogo Prefectural Nishinomiya Hospital, 13-9 Rokutanji-cho, Nishinomiya, Hyogo 662-0918, Japan; ^3^Department of Radiology, Hyogo Prefectural Nishinomiya Hospital, 13-9 Rokutanji-cho, Nishinomiya, Hyogo 662-0918, Japan

## Abstract

POEMS syndrome is a multisystem disorder characterized by polyneuropathy, organomegaly, endocrinopathy, monoclonal gammopathy, and skin changes. POEMS syndrome is a rare cause of refractory ascites. We report the case of a patient with POEMS syndrome presenting with massive ascites who was treated with very-low-dose lenalidomide and dexamethasone. A 57-year-old Japanese man was admitted to our hospital with pleural effusion, massive ascites, and leg edema. The diagnosis of POEMS syndrome was made based on the combination of the following findings: peripheral neuropathy, organomegaly, endocrinopathy, serum monoclonal protein elevation, skin changes, plasma VEGF elevation, and evidence of extravascular volume overload. Renal dysfunction induced by biopsy-proven renal involvement of POEMS syndrome was observed. Massive ascites of the patient dramatically diminished with long-time treatment of very-low-dose lenalidomide and dexamethasone. Lenalidomide seems to be a very promising therapy for POEMS syndrome presenting with extravascular volume overload such as edema, pleural effusion, and ascites. Very-low-dose lenalidomide might be effective especially for the patients with POEMS-related nephropathy.

## 1. Introduction

POEMS syndrome is a monoclonal plasma cell disorder characterized by the presence of peripheral neuropathy and one or more of the following features: osteosclerotic myeloma, Castleman's disease, increased serum levels of vascular endothelial growth factor (VEGF), organomegaly, endocrinopathy, edema, typical skin changes, and papilledema [1]. Patients often present with evidence of extravascular volume overload, such as edema, pleural effusion, and ascites [2]. POEMS syndrome is a rare cause of refractory ascites. We report the case of a patient with POEMS syndrome who presented with massive ascites as the first manifestation, in whom the ascites dramatically diminished with long-term treatment of very-low-dose lenalidomide and dexamethasone.

## 2. Case Presentation

**Figure 1:**
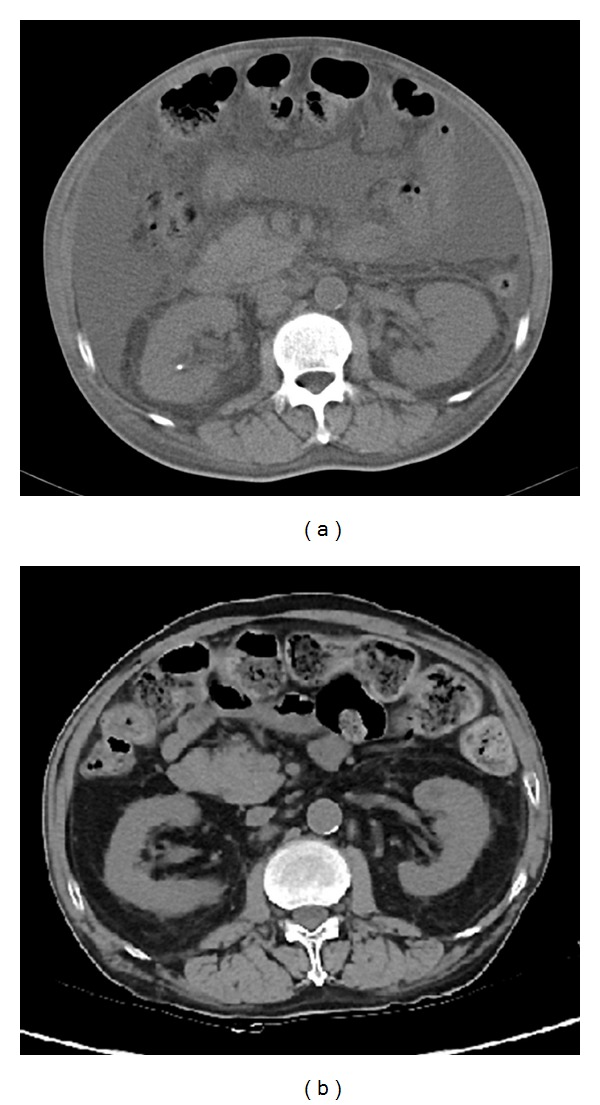
Computed tomographic scans of the abdomen. (a) Massive ascites before treatment. (b) Disappearance of ascites after treatment.

A 57-year-old Japanese man was admitted to our hospital with pleural effusion, massive ascites, and leg edema in March 2010. Physical examination revealed hyperpigmentation, hemangiomas, and gynecomastia. The patient had no neurological symptoms; however, the deep tendon reflexes were absent. Chest radiography revealed cardiomegaly and bilateral pleural effusion. Computed tomography of the abdomen revealed hepatosplenomegaly and massive ascites (Figure 1(a)). Blood examination showed moderate renal dysfunction (serum creatinine 1.81 mg/dL, blood urea nitrogen 44 mg/dL). The diagnosis of POEMS syndrome was made based on the following findings: peripheral neuropathy (mild decrease of the median nerve conduction velocity), organomegaly (hepatosplenomegaly), endocrinopathy (gynecomastia), serum monoclonal protein elevation (IgA lambda 1295 mg/dL), skin changes (hyperpigmentation and hemangioma), plasma VEGF elevation (700 pg/mL; normal <117 pg/mL), and evidence of extravascular volume overload (edema, pleural effusion, and massive ascites). While there were 5.4% plasma cells in bone marrow aspirate and biopsy, neoplastic plasma cells were not observed. The ratio of serum-free kappa and lambda light chain was within normal range. The diagnostic process of this patient was described in the previous report [3]. The patient had the renal involvement of POEMS syndrome and the renal biopsy was helpful for making a precise diagnosis [3].

Initially the patient refused medication for POEMS syndrome. However, the ascites increased gradually in size, necessitating regular drainage from July 2010, with the average drainage volume per session being 6000 mL. From December 2010, drainage was necessitated three times a month (average drainage volume: 18 liters/month).

The patient gave consent for undergoing treatment. We started treatment with lenalidomide (10 mg/day for 21 days of a 28-day cycle) and once-weekly dexamethasone (10 mg). While the ascites and plasma VEGF concentration reduced dramatically (VEGF: 64 pg/mL), the treatment could not be continued due to deterioration of the renal function. After discontinuation of the lenalidomide (once-weekly dexamethasone was continued to be administrated), the patient's renal function recovered to its pretreatment level; however, the ascites began to increase again in size. We then restarted the treatment with a lower dose of lenalidomide (5 mg every other day for 11 days of a 28-day cycle) and dexamethasone (10 mg/week). The ascites gradually began to reduce in size in response to this treatment. While the plasma level of VEGF increased transiently (244 pg/mL at 6 months after the retreatment), it decreased finally to be within normal range (73 pg/mL and 26 pg/mL at 12 and 18 months after the retreatment, resp.). By the end of the seventh course of retreatment, the ascites had completely disappeared (Figure 1(b)). The patient did not want to undergo high-dose chemotherapy with autologous stem cell transplantation as a consolidative therapy. At the time of writing, the patient remains on maintenance therapy with very-low-dose lenalidomide and dexamethasone and is free of ascites. The median nerve conduction velocity has increased slightly (about 10%) and the renal function improved (serum creatinine: 0.93 mg/dL, blood urea nitrogen: 13 mg/dL). Although the level of serum IgA is tending to decline, the pace of reduction is considerably slow (1002 mg/dL and 981 mg/dL at 12 and 18 months after the retreatment, resp.).

## 3. Discussion

While the most common cause of ascites is liver cirrhosis, other less frequently encountered causes include infection, cardiac disease, nephrotic syndrome, pancreatic disease, lymphatic obstruction or leakage, noncirrhotic portal hypertension, collagen disease, and peritoneal carcinomatosis. POEMS syndrome is a rarely encountered paraneoplastic syndrome that often manifests with features of extravascular volume overload. VEGF is thought to be responsible for the majority of the symptoms. Increased vascular permeability and angiogenesis lead to edema, pleural effusion, and ascites.

Antiangiogenic drugs, such as thalidomide and lenalidomide, have been reported to be effective for the treatment of POEMS syndrome [4–8]. There are also a few reports on the usefulness of bortezomib in patients with POEMS syndrome [9, 10]. However, peripheral neuropathy is a major feature of this syndrome, and treatment with both thalidomide and bortezomib is known to be associated with a high incidence of drug-induced peripheral neuropathy. Lenalidomide has the advantage of being both antiangiogenic and cytotoxic to monoclonal plasma cells and of being associated with a much lower risk of peripheral neuropathy. In fact, Tomás et al. reported that all of 10 patients with POEMS syndrome in their series treated with lenalidomide as salvage therapy showed favorable responses without any unmanageable adverse events [8].

In our patient, treatment with lenalidomide brought about dramatic reduction of the massive ascites associated with POEMS syndrome, as well as improvement of the peripheral neuropathy. This is the first report of lenalidomide treatment of POEMS syndrome associated with massive ascites. Thus lenalidomide seems to be a very promising therapy for POEMS syndrome.

In POEMS syndrome, renal involvement occurs in a considerable proportion of the patients. Because lenalidomide is a nephrotoxic drug, lenalidomide administration may cause the deterioration of renal function in the patients with POEMS-related nephropathy. In fact, with low-dose lenalidomide (10 mg/day) the patient faced deterioration of renal function and the therapy had to be interrupted. Then we chose very-low-dose lenalidomide (5 mg/every other day) which reduced renal toxicity and enabled keeping long-term treatment. For the patients with POEMS-related nephropathy very-low-dose lenalidomide could be effective to improve clinical conditions.

Interestingly, while the patient showed favorable response to lenalidomide, the reduction of the serum level of the monoclonal protein was considerably small and slow. Thus, the clinical response with lenalidomide might be apparently due less to a reduction in the monoclonal plasma cell population than to a reduction of the blood VEGF level. In most patients with POEMS syndrome who showed clinical improvement, substantial decrease of the VEGF level is reported to be observed [6]. These reports are consistent with the view that VEGF plays a critical role in the pathogenesis of POEMS syndrome. For POEMS patients, long-term treatment with antiangiogenic drugs may be required, monitoring VEGF levels.
